# Dramatic Effect of Botulinum Toxin Type A on Hypertrophic Scar: A Promising Therapeutic Drug and Its Mechanism Through the SP-NK1R Pathway in Cutaneous Neurogenic Inflammation

**DOI:** 10.3389/fmed.2022.820817

**Published:** 2022-03-03

**Authors:** Shunuo Zhang, Ke Li, Zhixi Yu, Jun Chai, Zheng Zhang, Yixin Zhang, Peiru Min

**Affiliations:** Department of Plastic and Reconstructive Surgery, Shanghai Ninth People's Hospital Affiliated to Shanghai Jiao Tong University School of Medicine, Shanghai, China

**Keywords:** hypertrophic scar, therapeutic drug, botulinum toxin type A, benign solid tumor, cutaneous neurogenic inflammation, SP-NK1R signaling pathway

## Abstract

**Background:**

Hypertrophic scar formation may be related to cutaneous neurogenic inflammation (CNI) through the substance P-neurokinin 1 receptor (SP-NK1R) signaling pathway. As a widely used drug in aesthetic clinical work, botulinum toxin type A (BTX-A) has a therapeutic effect on scars, but the actual mechanism remains unclear. This study aimed to clarify the potential mechanism by which BTX-A inhibits CNI in hypertrophic scars both *in vitro* and *in vivo*.

**Methods:**

Tissue samples were obtained from surgical excisions. Immunohistological analysis was used to locate SP in human hypertrophic scars and normal skin. RT-PCR and western blot analysis were used to evaluate the expression of collagens after SP/BTX-A treatment. A rabbit ear scar model was used to explore the *in vivo* effect of BTX-A on scar treatment.

**Results:**

SP and NK-1R were overexpressed in hypertrophic scars compared to normal skin tissues. Collagen secretion of hypertrophic scar-derived fibroblasts increased with increasing doses of SP. However, BTX-A may downregulate collagen expression through SP-NK1R pathway with or without the presence of SP inducing agent capsaicin. Meanwhile, SP inhibited the expression of NK-1R, and this inhibition was blocked by pretreatment with BTX-A. *In vivo*, intralesional BTX-A injection can also reduce the volume of scars and inhibit collagen secretion. Capsaicin may cause more severe scar manifestations, while the therapeutic effect of BTX-A remains.

**Conclusion:**

Our research confirms that CNI stimulates fibroblasts during scar formation, while BTX-A can reduce collagen secretion by inhibiting the SP-NK1R signaling pathway, thus identifying a novel therapeutic target for this benign solid skin tumor.

## Introduction

Hypertrophic scarring is a fibrous hyperproliferative disease secondary to skin injury characterized by excessive fibroblasts and extracellular matrix (ECM) deposition, which resembles the process of benign solid tumor formation (1, 2). It is considered to be a multifactorial condition, and a large number of studies have explored its exact pathogenesis from the perspective of genetics, biomechanics, metabolism, endocrinology and immunology. In recent years, neurogenic inflammation (NI) has been proven to contribute to the formation of hypertrophic scars (3, 4). NI refers to an inflammatory process due to acute injury characterized by the release of neuropeptides, especially substance P (SP), from sensory nerves (5).

After skin injury, a large number of macrophages and lymphocytes accumulate around the wound surface to initiate an inflammatory response, and fibroblasts subsequently secrete abundant disorderly arranged collagens, which eventually lead to scar formation (6). In the scar healing process, the mechanical force generated by wound contraction may stimulate mechanosensitive nociceptors on sensory fibers to induce the secretion of SP and calcitonin gene-related peptide (CGRP) from peripheral nerve endings, thereby upregulating the expression of TGF-β and NGF in a variety of cells, including skin fibroblasts (7). Moreover, the overexpression of TGF-β in scar tissue induces the expression of α-SMA, which contributes to wound contraction by myofibroblasts (8). Thus, the peripheral nerve endings secrete more SP and CGRP, forming a continuous self-feedback microenvironment for local inflammation (9).

SP is a neuropeptide that mediates angiogenesis, keratinocyte proliferation and fibrogenesis (10). It is coexpressed in the nerve fibers of human skin tissue with the transient receptor potential ion channel (TRPV1), which not only transmits pain signals but also regulates inflammatory responses to tissue injury (11). SP is selectively bound by its specific receptor neurokinin-1 receptor (NK-1R) (12). In recent years, many studies have shown that the SP-NK1R signaling pathway is highly related to the occurrence of chronic inflammation, wound healing and tumorigenesis (13). Researchers have found that SP is overexpressed in burn wounds and hypertrophic scars, while SP-positive nerve fibers are mainly distributed around blood vessels, hair follicles and sebaceous glands (14, 15). Our previous study proved that blocking peripheral nerve signal transmission around hair follicles can significantly inhibit hair follicle unit function, which further confirms that the neurotransmitter released by the peripheral nerve terminal is mainly distributed around hair follicles (16). Therefore, additional insight into the metabolic abnormalities mediated by fibroblasts will help clarify the role of cutaneous neurogenic inflammation (CNI) in hypertrophic scars.

Botulinum toxin type A (BTX-A) has become the most widely used drug in esthetic clinical settings. The drug is believed to block the presynaptic release of acetylcholine (ACh) by proteolytic cleavage of the SNARE family protein SNAP-25 (17). However, in recent years, several studies have found that BTX-A can also block the signal transmission of non-cholinergic neurotransmitters (18). Inhibition of the SP-NK1R signaling pathway has also been found in some inflammatory diseases, such as respiratory inflammation and bladder injury, yet the mechanism remains to be discovered (19, 20). Some researchers believe that BTX-A may downregulate the uptake of trophic molecules, which is necessary for the synthesis of SP and CGRP, resulting in the inhibition of NI (21, 22). There are also theories about BTX-A acting as an inhibitor of neurotransmitters in the synaptic cleft by cleaving SNAP-25 and finally blocking NI (23, 24). Our preliminary research found that BTX-A can significantly reduce the collagen content in the dermis of normal skin, change the skin mechanical parameters, and inhibit the function of local hair follicles (16). Nevertheless, the mechanism of how BTX-A reduces hypertrophic scar formation remains unclear. Herein, this study aimed to clarify whether the SP-NK1R pathway plays an important role in scar formation and whether BTX-A can inhibit CNI through this pathway, providing a novel therapeutic target for hypertrophic scars.

## Materials and Methods

### Patients and Samples

Small samples of normal human skin and hypertrophic scars were obtained from patients treated at the Department of Plastic and Reconstructive Surgery, Shanghai Ninth People's Hospital, Shanghai Jiao Tong University. This research was approved by the Independent Ethics Committee of Shanghai Ninth People's Hospital affiliated to Shanghai Jiao Tong University School of Medicine, and informed consent was obtained from all patients.

Between December 2019 and November 2020, 6 normal skin samples and 8 hypertrophic scar samples were obtained. All subjects were Chinese and aged from 9 to 43 years (Supplementary Table 1). Hypertrophic scars were defined as visible and elevated scars confined to the borders of a burn injury or trauma site without any spread into surrounding tissues. Only newly developed and proliferative hypertrophic scar tissue samples were included in this study. None of the patients had undergone radiotherapy or hormone therapy. Additionally, normal skin samples were obtained from patients during unrelated surgical operations, and there was no extra harm to the patients. After the outline was exactly landmarked along the edges of scars with methylene blue, the hypertrophic scars were carefully excised by one skilled plastic surgeon. Once the scar tissues were removed, all tissues were divided into four portions with a cross-shaped incision and collected in sterile tubes immediately after surgical removal. One portion was fixed with formalin, dehydrated in alcohol, and embedded in paraffin to make a tissue section with a thickness of ~5 μm. A second portion was kept in the refrigerator at 4°C until processing, usually within 6 h. The rest were immediately frozen in liquid nitrogen and stored at −80°C until use.

### Cell Isolation and Amplification

Human fibroblasts of normal skin and hypertrophic scars were isolated and cultured from human skin samples after surgery as previously described. Briefly, the epidermis and subcutaneous adipose tissue were first removed while the dermis was dissected into 1 mm^3^ pieces. All pieces were digested with 2 mg/mL collagenase type I (Invitrogen, Grand Island, USA) at 37°C for 3 h. A single cell suspension was obtained after removing undigested tissue residues through a 150-mesh sterile filter. After centrifugation, the cells were cultured in Dulbecco's modified Eagle's medium (DMEM; HyClone, USA) supplemented with 10% fetal bovine serum (FBS; Gibco, USA), 100 U/mL penicillin G and 100 mg/ml streptomycin (Gibco, USA) in a humidified incubator containing 5% CO_2_ at 37°C. The culture medium was changed every 3 days, and the cells were passaged when 80% confluent. Human fibroblasts between the third and fifth passages were used in the following experiments.

### SP/BTX-A/Capsaicin/Aprepitant Treatment

The culture medium in the petri dish was discarded. After rinsing gently with 3–4 mL sterile PBS solution 3 times, 1 mL 0.25% trypsin (Gibco, USA) was added to the petri dish and transferred to a 37°C incubator for 1–2 min. When the adherent cells turned round, 2.5 mL of serum-containing culture medium was added to the culture dish to stop the digestion, and then we gently pipetted to form a cell suspension. After that, the cell suspension was transferred to a sterile tube and centrifuged for 1,500 rpm for 5 min. The supernatant was discarded, and an appropriate amount of culture medium was added to the tube. A small amount of cell suspension was then taken to make a smear and quickly dried in cold air. The fibroblasts were counted, and the cell concentration was adjusted to 5 × 10^5^ cells/mL. Two milliliters of the cell suspension were inoculated per well in a 12-well culture plate and then incubated for 12 h with 5% CO_2_ at 37°C.

SP (Abcam, ab120170) was added at concentrations of 1, 10, and 100 ng/mL to each well in the culture medium with normal saline as a blank control group. Then, it was incubated for 2 or 4 h, respectively. 4 U/mL or 10 U/mL of BTX-A (Botox, Allergan Inc., Irvine CA, USA) and 25 μM/L capsaicin (Abcam, ab141000) were introduced separately or in combination to each well of the culture medium and then incubated for 4 h. After pretreatment with aforementioned two doses of BTX-A for 2 h, 1, 10, or 100 ng/mL SP with or without 25 μM/L capsaicin was added to each well of the culture medium and then incubated for 4 h. Five micrometer specific NK-1R antagonist aprepitant (MedChemExpress, CAS No. 170729-80-3) was applied to detect the targeting effect of BTX-A on SP-NK1R pathway.

### Protein Extraction and Western Blot

Total cellular proteins were extracted using RIPA lysis buffer containing phosphatase and protease inhibitors (Beyotime Biotechnology, China). The concentration of total protein was detected with a BCA Protein Assay kit (Beyotime Biotechnology, China). Equal amounts (20 μg) of protein were separated using 4–20% SDS-PAGE gels. The proteins were then transferred to nitrocellulose membranes (0.45 μm; Millipore, USA). The membranes were blocked with 5% non-fat milk for 1 h at room temperature and incubated with primary antibodies (Table 1) overnight at 4°C. After washing, the membranes were incubated with goat anti-rabbit secondary antibodies (Table 1). Finally, the membranes were visualized using the Odyssey CLx Infrared Imaging System (LI-COR Biosciences, Lincoln, Nebraska, USA). *GAPDH* and β-actin protein intensities were used as internal controls.

**Table 1:** List of antibodies.

**Primary antibodies**	
*GAPDH*	Abcam, ab8245, 36 KDa, mouse, 1:2000
β-*Actin*	Abcam, ab822, 42 KDa, mouse, 1:2000
*NK-1R*	Affnity, DF4996, 50 or 37 KDa, rabbit, 1:1000
*CGRP*	Affnity, DF7785, 15 KDa, rabbit, 1:1000
*SP*	Affnity, DF6541, 15 KDa, rabbit, 1:1000
*COL1*	Servise, GB11022-2, 110–130 KDa, rabbit, 1:1000
*COL3*	Servise, GB11023, 143 KDa, rabbit, 1:1000
**Secondary antibodies**	
goat anti-mouse IgG	Abcam, ab205719, goat, 1:5000
goat anti-rabbit IgG	Abcam, ab6721, goat, 1:5000

### RNA Extraction and Real-Time Reverse-Transcriptase Polymerase Chain Reaction

Total RNA was extracted using TRIzol reagent (Invitrogen, USA) according to the manufacturer's protocol. Then, 1 μg of total RNA was reverse transcribed into cDNA using the FastKing cDNA synthesis kit (Tiangen Biotech, China). qPCR was performed with TB Green Premix (Takara, Japan). The mRNA expression levels of *NK-1R, CGRP, SP*, type 1 and 3 collagen were detected. PCR primers were designed based on sequences from the corresponding genes (Table 2). All data were normalized using *GAPDH* as the internal control by the ΔCT method.

**Table 2:** The primers for Real-Time PCR.

**Primers**	
**Human**
*GAPDH F*	5′-GGAGCGAGATCCCTCCAAAAT-3′
*GAPDH R*	5′-GGCTGTTGTCATACTTCTCATGG-3′
*NK-1R F*	5′-TGATGTGGATCATCTTAGCCCA-3′
*NK-1R R*	5′-GAACAGGCCGTAGTACCATTC-3′
*CGRP F*	5′-ATGCAGCACCATTCAGGTCTG-3′
*CGRP R*	5′-CCAGCCGATGAGTCACACAG-3′
*SP F*	5′-ACTGGTCCGACTGGTACGACA-3′
*SP R*	5′-AAGAACTGCTGAGGCTTGGGT-3′
*COL1 F*	5′-ATTGCCTTTGATTGCTGGGCAGAC-3′
*COL1 R*	5′-CAATGCTGCCCTTTCTGCTCCTTT-3′
*COL3 F*	5′-GCCAAATATGTGTCTGTGACTCA-3′
*COL3 R*	5′-GGGCGAGTAGGAGCAGTTG-3′
**Rabbit**
β-Actin F	5′-GCAGAAACGAGACGAGATTG-3′
β-Actin R	5′-GCAGAACTTTGGGGACTTTG-3′
*SP F*	5′-AGGGCTGAAATGTACTGAGTC-3′
*SP R*	5′-GAACCACAAACCGTGAAAC-3′

### Animals and Wound Model

Rabbit hypertrophic scar models were constructed for the *in vivo* studies with approval from the Animal Experimentation Ethics Committee of the School of Medicine, Shanghai Jiao Tong University. We injected 100 mg/kg capsaicin (Abcam, ab141000) subcutaneously into 6 animals (5 mL per day on 2 consecutive days before surgery) in the dorsal thoracic region under light inhalation anesthesia (3–5% sevoflurane).

A total of 12 adult New Zealand white rabbits (2.0–2.5 kg, Si-Lai-Ke, Shanghai, China) were anesthetized using pentobarbital sodium delivered intravenously via ear veins (30 mg/kg). On the ventral surface of each ear, four wounds (10 mm diameter) were created down to bare cartilage, and the perichondrium was removed. After the surgery, the rabbits were allowed to move freely in individual cages and consume standard food. The wounds were covered using sterile gauze for 1 day. After 4 weeks, when the scar was formed, each rabbit ear was randomly divided into groups. Then, 2 U, 4 U, or 10 U BTX-A was injected into the scars in an intralesional manner, while normal saline was injected as a blank control. The samples were taken 3 or 6 weeks after BTX-A injection.

### Histological Analysis

#### Immunohistochemistry

The expression of SP in human hypertrophic scars and normal skin was examined using an anti-SP antibody (Abcam, ab133240) by a standard immunohistochemistry protocol. In short, dewaxed sections were incubated with primary anti-SP antibody (Abcam, ab133240) at a dilution of 1:300 for 1 h at room temperature and then incubated with goat anti-rabbit immunoglobulin (HRP, Abcam, ab205718) secondary antibody for 30 min at room temperature. The levels of SP expression in the scar tissues were then compared with those in normal skin samples. The analysis of PCNA was performed in the same way using an anti-PCNA antibody (Abcam, ab92552).

#### Immunofluorescence

Standard immunofluorescence for TRPV1 (Abcam, ab3487) was performed on collected human tissue samples. After being deparaffinized and rehydrated, it was subjected to EDTA antigen retrieval solution (Servicebio Science, China), and the sample was blocked by H_2_O_2_ and serum. Then, the samples were coincubated with primary rabbit antibody against human TRPV1 overnight at 4°C, covered with secondary goat anti-rabbit antibody (Alexa Fluor^®^ 488, Abcam, ab150077) and incubated for 45 min in the dark at 37°C. 4′,6-diamidino-2-phenylindole (DAPI; blue emission signal) was applied to stain nuclei. The expressions of TRPV1 (green emission signal) were then observed with an inverted immunofluorescence microscope (Nikon DS-U3, Nikon, Japan).

### Statistical Analysis

Data analysis was performed using GraphPad Prism (8.0.2, America). Continuous variables are presented as the mean (±standard deviation, SD). Independent Student's *t*-test was applied to compare the means for two groups. One-way analysis of variance (ANOVA) was performed to compare the means of multiple groups, and if significant, a *post hoc* Tukey's test was used to explore the pairwise differences between groups. *P*-values < 0.05 were considered statistically significant.

## Results

### Expression and Localization of SP and TRPV1 in Human Hypertrophic Scar and Normal Skin Tissue Samples

**Figure 1:**
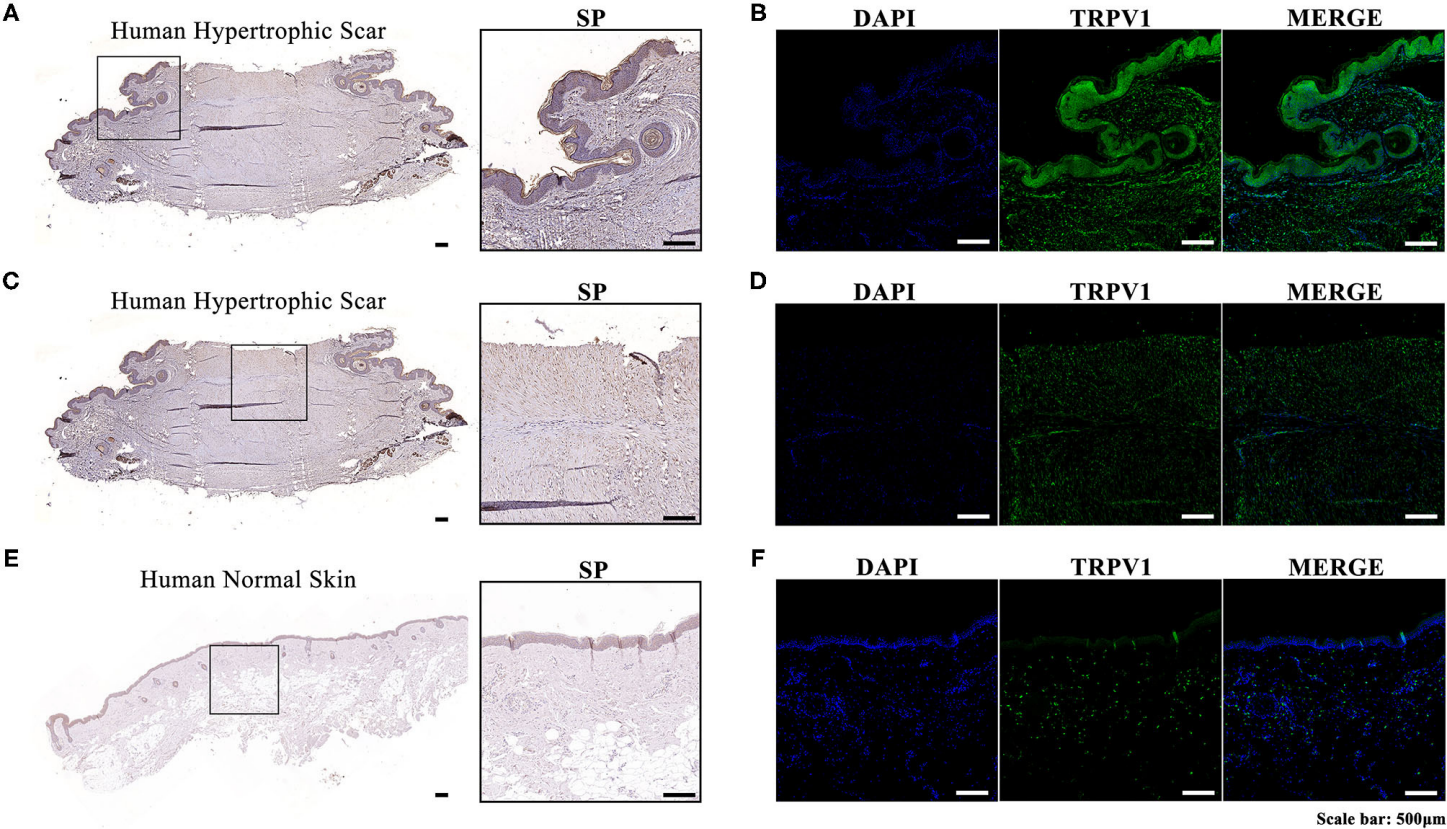
**(A)** Immunohistochemistry of the expression and localization of SP along the margin and around the follicles in a human hypertrophic scar tissue sample; **(B)** Immunofluorescence of the expression and localization of TRPV1 along the margin and around the follicles of a human hypertrophic scar tissue sample (left, DAPI; middle, TRPV1; right, MERGE); **(C)** Immunohistochemistry of the expression and localization of SP in the center of a human hypertrophic scar tissue sample; **(D)** Immunofluorescence of the expression and localization of TRPV1 in the center of a human hypertrophic scar tissue sample (left, DAPI; middle, TRPV1; right, MERGE); **(E)** Immunohistochemistry of the expression and localization of SP in a human normal skin tissue sample; **(F)** Immunofluorescence of the expression and localization of TRPV1 in a human normal skin tissue sample (left, DAPI; middle, TRPV; right, MERGE).

We performed immunohistochemistry and immunofluorescence in both human hypertrophic scar and normal skin tissue samples. The expression levels of SP and TRPV1 in human hypertrophic scar samples were significantly higher than those in normal skin samples. Moreover, we noticed that SP was overexpressed along the margin of the hypertrophic scar sample and around the follicles. Meanwhile, immunofluorescence showed the distribution of TRPV1 aligned with that of SP, which verified the overexpression of SP in hypertrophic scar tissue (Figure 1).

### Expression of SP, CGRP, and NK-1R in Human Hypertrophic Scar and Normal Skin Tissue Samples

**Figure 2:**
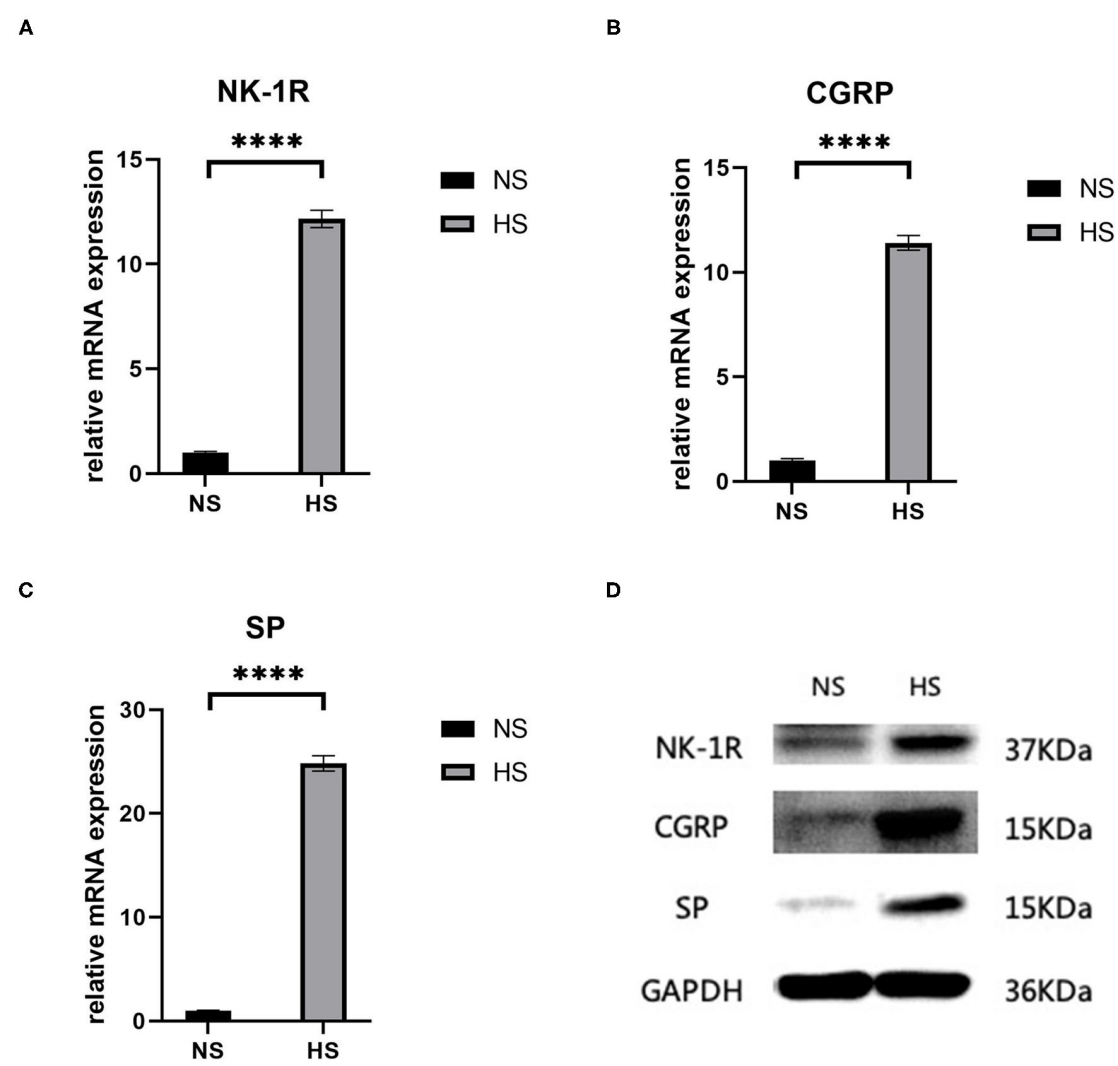
**(A)** Relative mRNA expression of *NK-1R* in human hypertrophic scar and normal skin tissue samples; **(B)** Relative mRNA expression of *CGRP* in human hypertrophic scar and normal skin tissue samples; **(C)** Relative mRNA expression of *SP* in human hypertrophic scar and normal skin tissue samples; **(D)** Expression of NK-1R, CGRP, and SP in human hypertrophic scar and normal skin tissue samples detected by western blot analysis. The results are represented as the mean of 3 experiments ± SD, which were performed in duplicate for each experimental condition. NS, normal skin; HS, hypertrophic scar (****P < 0.0001).

Both RT-PCR and western blot showed a significant increase (*p* < 0.0001) in the concentration of SP-related mRNA and proteins (SP, CGRP, NK-1R) in hypertrophic scar tissue compared to the normal samples (Figure 2). This confirms the role of SP along with its coproteins in the process of hypertrophic scar formation.

### SP Upregulates the Collagen Expression of Hypertrophic Scar-Derived Fibroblasts

**Figure 3:**
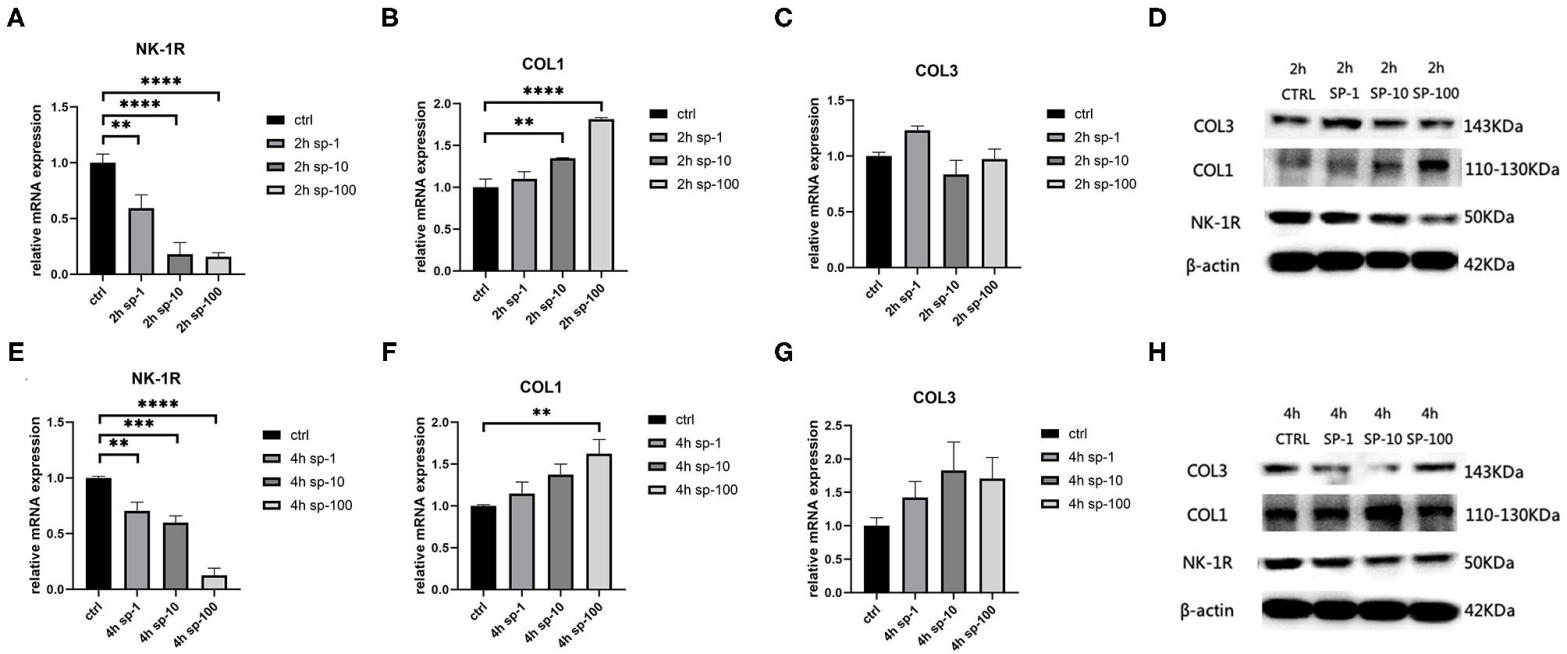
The mRNA and protein expressions were from hypertrophic scar-derived fibroblasts treated with increasing concentrations of SP. **(A)** Relative mRNA expression of *NK-1R* 2 h after treatment with 1, 10, and 100 ng/ml of SP; **(B)** Relative mRNA expression of type 1 collagen 2 h after treatment with 1, 10, and 100 ng/mL of SP; **(C)** Relative mRNA expression of type 3 collagen 2 h after treatment with 1, 10, and 100 ng/mL of SP (ANOVA showed no significance); **(D)** Expression of NK-1R, type 1 and 3 collagen 2 h after treatment with 1, 10, and 100 ng/mL of SP detected by western blot analysis; **(E)** Relative mRNA expression of *NK-1R* 4 h after treatment with 1, 10, and 100 ng/mL of SP; **(F)** Relative mRNA expression of type 1 collagen 4 h after treatment with 1, 10, and 100 ng/mL of SP; **(G)** Relative mRNA expression of type 3 collagen 4 h after treatment with 1, 10, and 100 ng/mL of SP (ANOVA showed no significance); **(H)** Expression of NK-1R, type 1 and 3 collagen 4 h after treatment with 1, 10, and 100 ng/mL of SP detected by western blot analysis. The results are represented as the mean of 3 experiments ± SD, which were performed in duplicate for each experimental condition (**P < 0.01; ***P < 0.001; ****P < 0.0001).

After 2 h of SP treatment, we noticed that the relative mRNA expression of *NK-1R* decreased significantly as the concentration of SP increased (*p* < 0.001), and this also occurred after 4 h of SP treatment (*p* < 0.001) (Figures 3A,E). Moreover, as the concentration of SP increased, the expression of *NK-1R* decreased significantly, indicating that the expression of *NK-1R* was negatively correlated with the concentration of SP. Meanwhile, after 2 h of SP treatment, the concentration of type 1 collagen increased significantly (*p* < 0.001), while type 3 collagen seemed stable without significant changes (*p* > 0.05) (Figures 3B,C). After 4 h of SP treatment, the concentration of type 1 collagen also increased (*p* < 0.01), while type 3 collagen remained unchanged (*p* > 0.05) (Figures 3F,G). Likewise, the expression of type 1 collagen increased with increasing doses of SP, indicating a positive correlation of the expression of type 1 collagen with the concentration of SP. Western blot analysis showed a similar tendency in terms of proteins (Figures 3D,H).

### BTX-A Inhibits the Expression of SP in Hypertrophic Scar-Derived Fibroblasts

**Figure 4:**
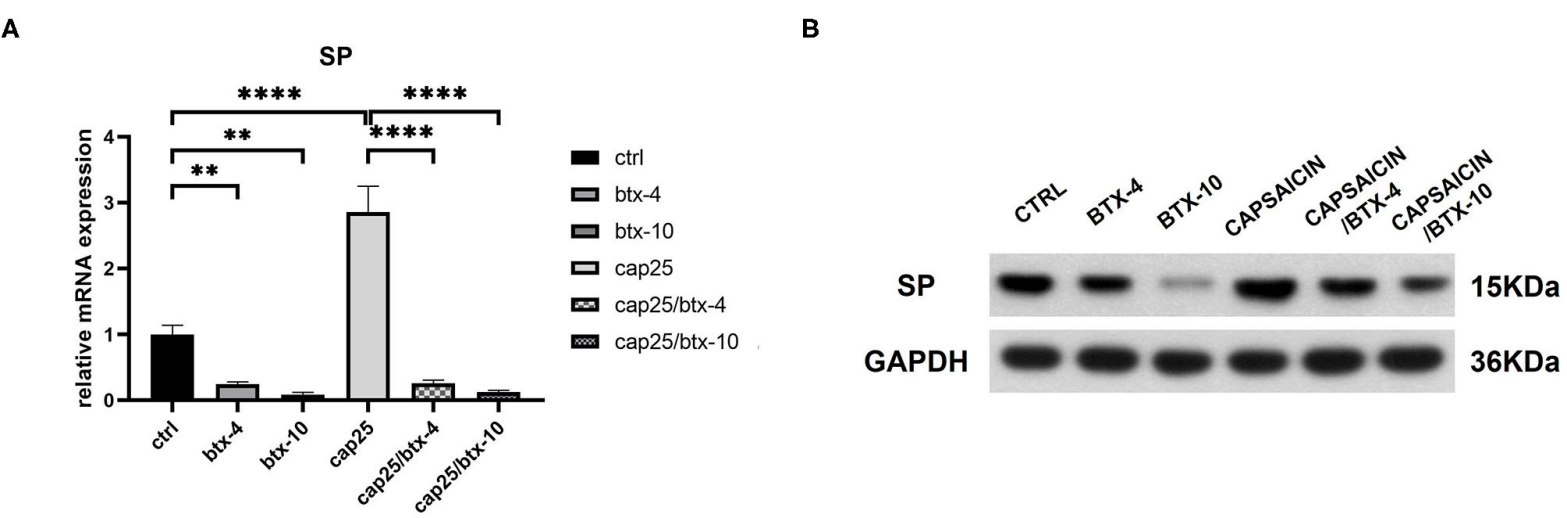
The mRNA and protein expressions were from hypertrophic scar-derived fibroblasts treated with BTX-A (4 or 10 U/mL) and capsaicin (25 μM/L). **(A)** Relative mRNA expression of *SP* after treatment with BTX-A and capsaicin separately or in combination; **(B)** Expression of SP after same BTX-A and capsaicin treatment detected by western blot analysis. The results are represented as the mean of 3 experiments ± SD, which were performed in duplicate for each experimental condition (**P < 0.01; ****P < 0.0001).

To determine the inhibitive effect of BTX-A on SP, we treated human hypertrophic scar-derived fibroblasts with BTX-A and capsaicin separately or in combination. The RT-PCR showed that capsaicin (25 μM/L) significantly induce the *SP* expression while BTX-A (4 U/mL or 10 U/mL) significantly reduce the *SP* expression (Figure 4A). Interestingly, even with the presence of capsaicin, the expression of *SP* remained inhibited by BTX-A (Figure 4A). The similar results were observed on western blot (Figure 4B).

### Pretreatment With BTX-A Downregulate the Collagen Expression of Hypertrophic Scar-Derived Fibroblasts

**Figure 5:**
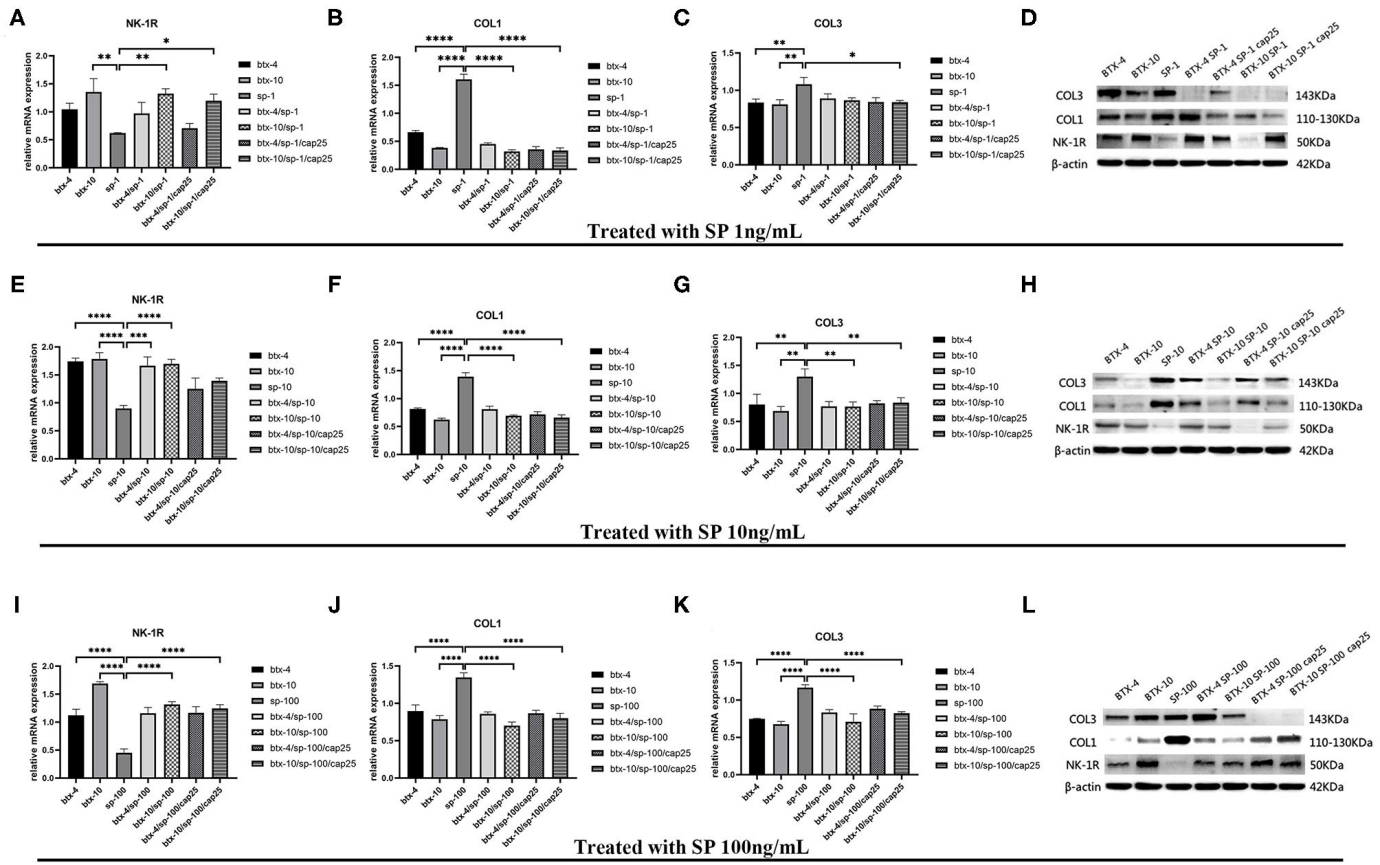
The mRNA and protein expressions were from hypertrophic scar-derived fibroblasts treated with increasing concentrations of SP. **(A–C)** Relative mRNA expression of *NK-1R*, type 1 and 3 collagen after treatment with 1 ng/ml SP alone, 4 or 10 U/mL BTX-A with or without 1 ng/mL SP and 25 μM/L capsaicin; **(D)** Expression of NK-1R, type 1 and 3 collagen 2 h after treatment with 1 ng/ml SP alone, 4 or 10 U/mL BTX-A with or without 1 ng/mL SP and 25 μM/L capsaicin detected by western blot analysis; **(E–G)** Relative mRNA expression of *NK-1R*, type 1 and 3 collagen after treatment with 10 ng/ml SP alone, 4 or 10 U/mL BTX-A with or without 10 ng/mL SP and 25 μM/L capsaicin; **(H)** Expression of NK-1R, type 1 and 3 collagen 2 h after treatment with 10 ng/ml SP alone, 4 U/mL or 10 U/mL BTX-A with or without 10 ng/mL SP and 25 μM/L capsaicin detected by Western blot analysis; **(I–K)** Relative mRNA expression of *NK-1R*, type 1 and 3 collagen after treatment with 100 ng/ml SP alone, 4 or 10 U/mL BTX-A with or without 100 ng/mL SP and 25 μM/L capsaicin; **(L)** Expression of NK-1R, type 1 and 3 collagen 2 h after treatment with 100 ng/mL SP alone, 4 or 10 U/mL BTX-A with or without 100 ng/mL SP and 25 μM/L capsaicin detected by western blot analysis. The results are represented as the mean of 3 experiments ± SD, which were performed in duplicate for each experimental condition (*P < 0.05; **P < 0.01; ***P < 0.001; ****P < 0.0001).

Based on the above results, we conducted further experiments to explore the effect of BTX-A on collagen expression in hypertrophic scar-derived fibroblasts. The fibroblasts with no treatment were considered as the control group. We found that when human hypertrophic scar-derived fibroblasts were treated with BTX-A without induction of SP, type 1 collagen presented a significant decrease with elevated doses of BTX-A. Meanwhile, both low-dose and high-dose BTX-A successfully suppressed the overexpression of type 1 collagen induced by SP (*p* < 0.0001). Furthermore, this effect remained in the presence of the SP inducing agent capsaicin. Regardless of the dose of SP (1, 10, and 100 ng/ml) we used for stimulation, the expression of type 1 collagen remained reduced. Type 3 collagen presented a similar tendency as type 1 collagen but with less significance. The expression of NK-1R was increased with BTX-A pretreatment, but it seemed to be decreased in the presence of SP and capsaicin (Figure 5).

### Evidence That BTX-A Mediates Collagen Downregulation Is Blocked by NK-1R Antagonist

**Figure 6:**
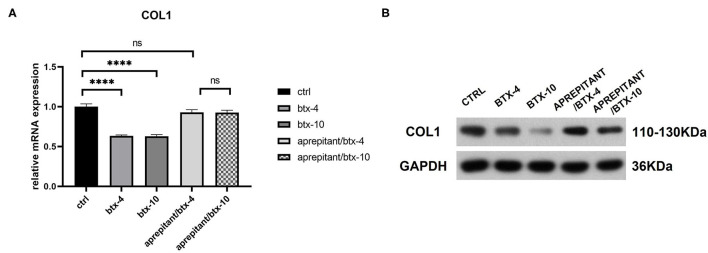
The mRNA and protein expressions were from hypertrophic scar-derived fibroblasts treated with BTX-A (4 or 10 U/mL) and NK-1R antagonist aprepitant (5 μM). **(A)** Relative mRNA expression of type 1 collagen after treatment with BTX-A in the presence or absence of aprepitant; **(B)** Expression of type 1 collagen after same BTX-A and aprepitant treatment detected by western blot analysis. The results are represented as the mean of 3 experiments ± SD, which were performed in duplicate for each experimental condition (ns *P* ≥ 0.05; ****P < 0.0001).

We treated human hypertrophic scar-derived fibroblasts with BTX-A in the presence or absence of NK-1R antagonist aprepitant to detect the targeting effect of BTX-A on SP-NK1R pathway. We noticed that the expression of type 1 collagen was significantly reduced by 4 or 10 U/mL BTX-A. However, when aprepitant (5 μM) was applied, the expression of type 1 collagen after BTX-A treatment showed no significant difference with the untreated control group (BTX-4/aprepitant, *p* = 0.1856 and BTX-10/aprepitant, *p* = 0.1634) (Figure 6). Hence, we concluded that SP-NK1R pathway mediates BTX-A to downregulate collagen expression in hypertrophic scars.

### Upregulation of SP Expression After Subcutaneous Injection of Capsaicin

**Figure 7:**
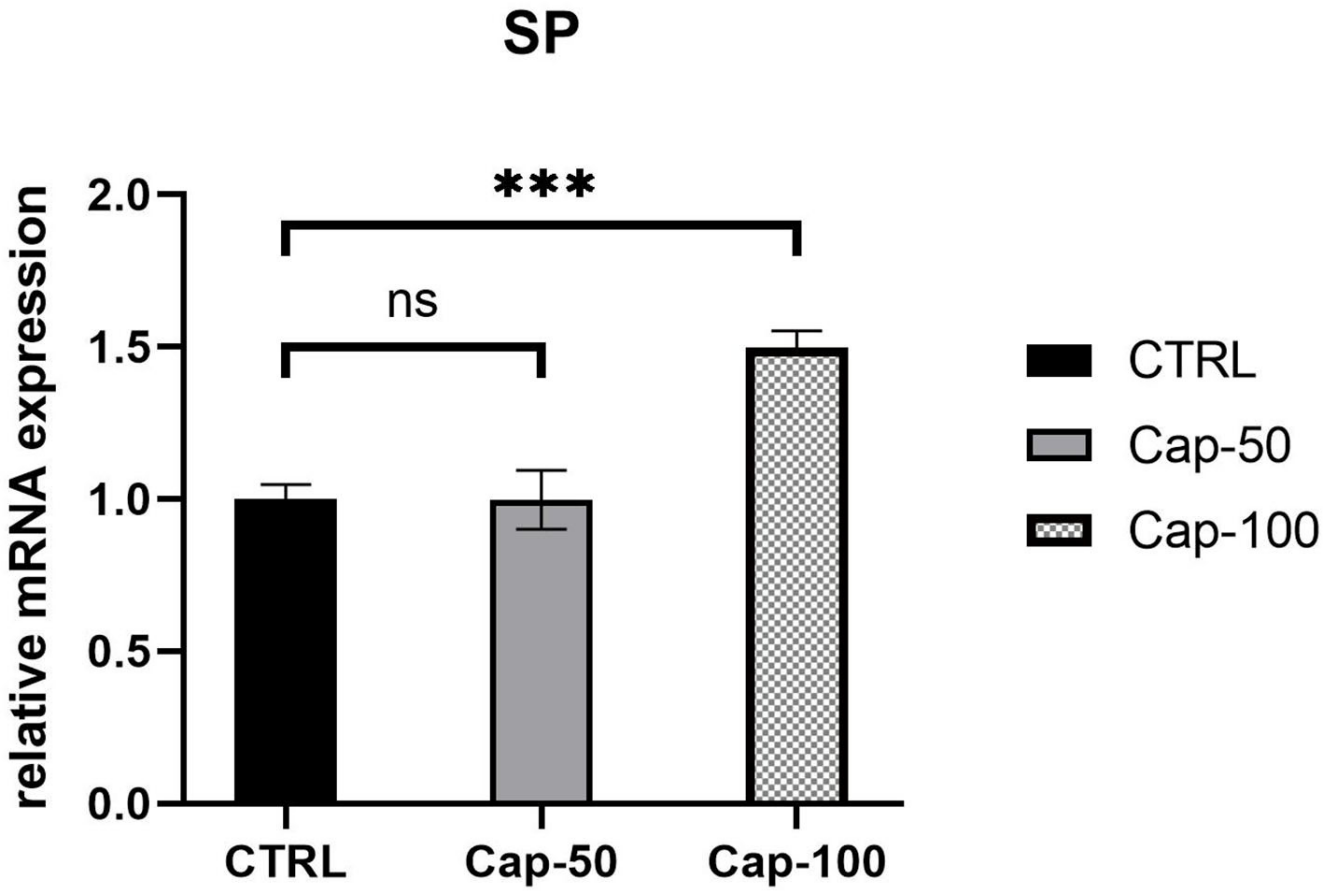
Relative mRNA expression of serum *SP* 1 week after the subcutaneous injection of 50 or 100 mg/kg capsaicin. The results are represented as the mean of 3 experiments ± SD, which were performed in duplicate for each experimental condition (ns *P* ≥ 0.05; ***P < 0.001).

*In vivo*, we performed subcutaneous injections of capsaicin to construct the capsaicin-induced SP overexpression model. At the end of the first week, we noted a significant increase in the serum *SP* expression of mRNA in the high-dose group (100 mg/kg) (*p* < 0.001). In the low-dose group (50 mg/kg), *SP* seemed unchanged compared to the control group (*p* ≥ 0.05) (Figure 7). Thus, we chose 100 mg/kg to establish the SP overexpression model in subsequent *in vivo* studies.

### *In vivo* Animal Model Results

**Figure 8:**
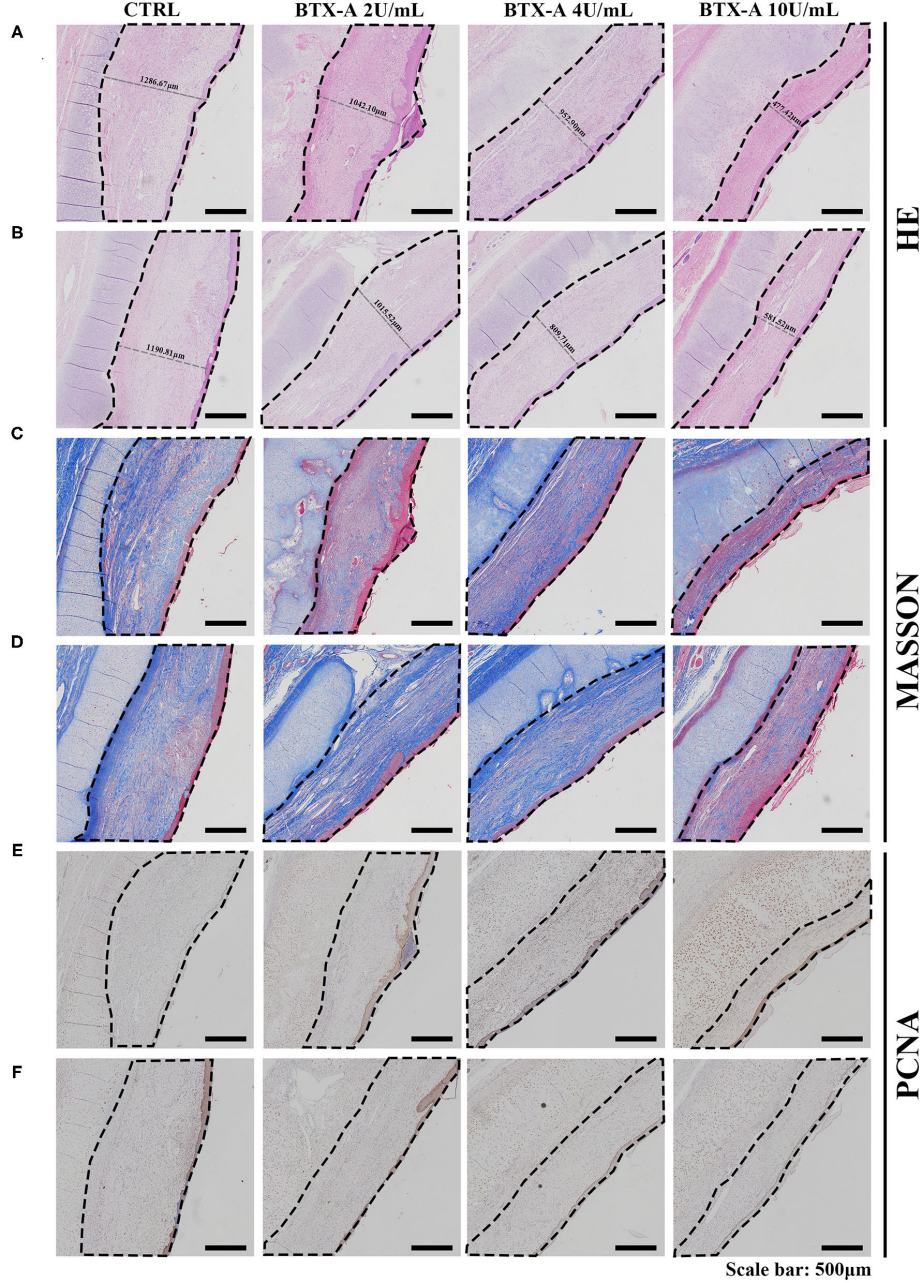
Results at 3 weeks after the treatment. The scar tissues were marked with dotted lines. **(A)** HE staining of different treated scar samples in the SP overexpression group; **(B)** HE staining of different treated scar samples in the SP normal-expression group; **(C)** Masson trichrome staining of different treated scar samples in the SP overexpression group; **(D)** Masson trichrome staining of different treated scar samples in the SP normal-expression group; **(E)** PCNA of different treated scar samples in the SP overexpression group; **(F)** PCNA of different treated scar samples in the SP normal-expression group.

Three weeks after the injection of normal saline and different concentrations of BTX-A, we collected histological samples from rabbits to analyze their scar tissue. The results of 4 different treatment subgroups and 2 different SP expression groups indicated that, compared with the normal SP expression group, the SP overexpression group showed a significant increase in thickness when treated with normal saline (Figures 8A,B). For both the SP overexpression and normal-expression groups, within the 4 BTX-A treatment subgroups, we found that the higher the dose of BTX-A was, the more regression of scar thickness was witnessed (Figures 8A,B). In addition, Masson trichrome staining revealed less collagen deposition in the BTX-A-treated groups, while the thickness was related to the concentration of BTX-A (Figures 8C,D). The PCNA analysis also revealed less cell mobilization in the BTX-A-treated groups (Figures 8E,F).

**Figure 9:**
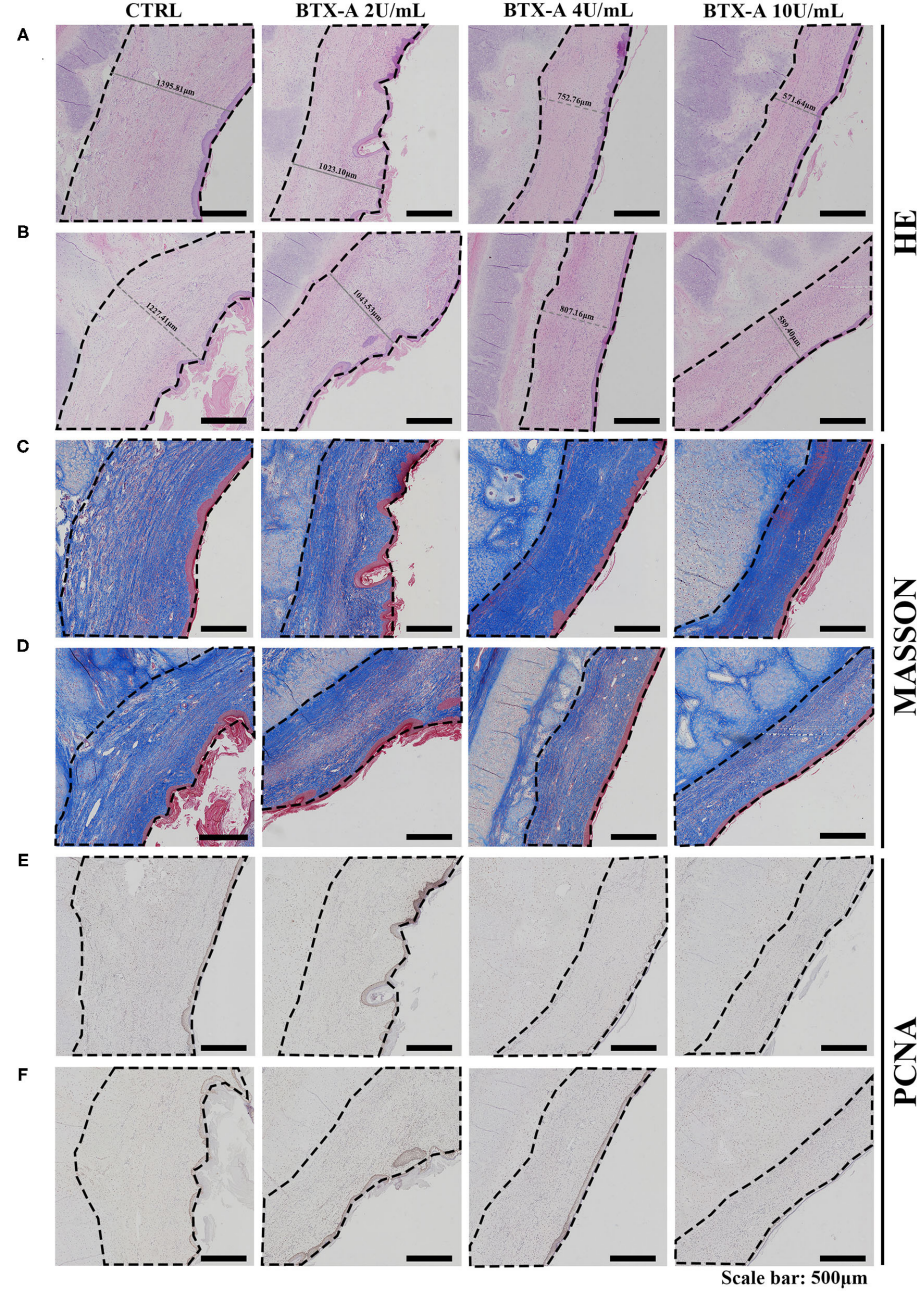
Results at 6 weeks after the treatment. The scar tissues were marked with dotted lines. **(A)** HE staining of the different treated scar samples in the SP overexpression group; **(B)** HE staining of the different treated scar samples in the SP normal-expression group; **(C)** Masson trichrome staining of the different treated scar samples in the SP overexpression group; **(D)** Masson trichrome staining of the different treated scar samples in the SP normal-expression group; **(E)** PCNA of the different treated scar samples in the SP overexpression group; **(F)** PCNA of the different treated scar samples in the SP normal-expression group.

We also collected histological scar samples from rabbits 6 weeks after the injection of normal saline and different concentrations of BTX-A. The results of different subgroups groups indicated that the scar tissues were thicker in the SP overexpression group than in the normal SP expression group (Figures 9A,B). The thickness of the scar tissues was also related to the dose of BTX-A (Figures 9A,B). However, Masson trichrome staining and PCNA analysis showed fewer differences between the two groups after 6 weeks (Figures 9C–F).

## Discussion

As benign solid skin tumors, treating or preventing hypertrophic scarring is a tough challenge without any specific therapy that has troubled surgeons for decades. A growing number of studies have been conducted in recent years to explore the existence of CNIs in various skin disorders and tumors, such as pruritus, psoriasis and esophageal carcinoma (9, 12). Clinically, hypertrophic scars in the proliferative stage often present typical symptoms related to inflammation, including erythema, itching and pain, which coincide with the manifestation of neurogenic inflammation and are characterized by antidromic stimulation of sensory fibers as well as activation of neuropeptides in skin inflammation (4, 25). Interestingly, these symptoms tend to be relieved or disappear once entering the stable stage; thus, we assume that CNI plays an important role in the development of hypertrophic scars.

As previously reported, the SP-NK1R pathway has been proven to be one of the most important signaling pathways in CNI (11–13). SP is an important regulatory peptide in the process of CNI and has been discovered to be overexpressed in several inflammatory skin diseases (14). NK-1R is a 7-fold G protein-coupled receptor that is widely distributed in the human body and is an important mediator in the process of cell proliferation, pain, inflammation, depression, cancer progression, etc. (26–28). NK-1R was reported to combine with SP and generate subsequent vasodilator properties as well as mobilize immune cells in the context of topical inflammation (29, 30). Studies have found the abnormal presence of SP and NK-1R in tumors such as esophageal and colon cancers, while SP and NK-1R also show abnormal expression in burn wounds and scars (12–15, 25, 31, 32). Therefore, we hypothesize that the existence of CNI is correlated with the formation of hypertrophic scars and that its intensity corresponds to the severity of clinical symptoms. As the SP-NK1R signaling pathway plays an important role during this process, blocking the SP-NK1R signaling pathway may interfere with CNI, thus presenting a novel therapeutic target for the treatment of hypertrophic scars.

To verify the hypothesis stated above, we collected fresh human hypertrophic scar (HS) tissue and normal skin tissue to compare the differences in the expression of SP. The results demonstrated a significant overexpression of SP, especially around the hair follicles and in the marginal area of HS, which confirms the hypothesis of SP overexpression in proliferative stage scars. Other studies have also found that on account of abundant peripheral nerve terminals around hair follicles, some fibrotic diseases may cause excessive secretion of SP or abnormal expression of NK-1R around hair follicles, which is consistent with our results (33). Moreover, in hypertrophic scar tissue of the same origin, the overexpression of SP is more prominent in the marginal area and around hair follicles.

Interestingly, discrepancies in appearance and symptoms can often be noticed clinically in the same hypertrophic scar, with marginal tissue presenting more severe itching and pain as well as a tendency of outward expansion, while the central area of the scar tends to be less inflammatory, which is also consistent with our results. This eventually corresponds to increased levels of collagens in the edge of the scar tissue compared to the scar center (34). Furthermore, we demonstrated the overexpression of SP and NK-1R in human hypertrophic scars compared to normal skin at both the mRNA and protein levels. Thus, we confirmed that SP is widely distributed in hypertrophic scar tissue and that its specific receptor NK-1R is possibly expressed in the membrane of hypertrophic scar-derived fibroblasts. In conclusion, the SP-NK1R signaling pathway plays an important role in the formation of hypertrophic scars.

The final tumor-like appearance of hypertrophic scars is attributed to abnormal collagen metabolism, and fibroblasts have been proven to mainly induce the secretion of type 1 and 3 collagens in HS (35). Although we have proven the existence of the SP-NK1R signaling pathway, the specific mechanism by which the SP-NK1R pathway mediates the abnormal metabolism of fibroblasts remains unclear. Based on the results of the first part of our study, we assumed that as an important mediator during the process of CNI, a localized concentration of SP may be related to abnormal collagen metabolism in this area. To prove our hypothesis, we designed a concentration gradient of SP to stimulate hypertrophic scar-derived fibroblasts to evaluate its effect on collagen metabolism as well as the expression of NK-1R. We found that the mRNA and protein expression of type 1 collagen increased with increasing doses of SP, confirming the promoting effect of SP on the development of hypertrophic scars. However, the expression of type 3 collagen remained no significant changes. The results also showed that when we stimulated fibroblasts with increasing doses of SP, the mRNA and protein expression of NK-1R tended to decrease accordingly. The mechanism of this phenomenon remains unknown. Due to the G-protein coupled receptor NK-1R possesses 2 isoforms, full-length NK1R-FL and truncated C-terminal NK1R-Tr, a previous study demonstrated that the isoform NK1R-Tr exhibits less binding affinity to SP and less downstream signal activation (36). In addition, several studies have demonstrated that NK-1R may be degraded in response to the stimulation of SP or other agonists via receptor desensitization (37, 38). Therefore, we assume that with an “overdose” of SP binding to NK-1Rs, the detectable free NK-1R on the surface of the fibroblast membrane decreases significantly. Herein, we underscored the role of the SP-NK1R signaling pathway in the metabolism of proliferative scar collagens, providing a feasible novel therapeutic target for pathological scars.

On this basis, we pretreated these fibroblasts with different doses of BTX-A to explore its inhibitory effect on the SP-NK1R signaling pathway. BTX-A was found to present a direct inhibitive effect on SP expression. The results also indicated a significant decrease in the concentration of type 1 and 3 collagens after pretreatment with BTX-A. Type 1 and 3 collagens are major extracellular matrix components that are predominant in hypertrophic scar tissue compared to other types of collagens (39, 40). A previous study revealed that BTX-A was able to suppress the synthesis of both type 1 and 3 collagens by suppressing the TGF-β1 signaling pathway and activating matrix metalloproteinases (41). However, detailed explanations of the mechanism of BTX-A in this situation remain unclear, and further research needs to be carried out. According to our observation, the downregulation of collagen mediated via BTX-A may be blocked by antagonist of NK-1R, a highly specific receptor for SP. Therefore, we believe that BTX-A is able to downregulate the metabolism of hypertrophic scar-derived fibroblasts, confirming the role of BTX-A in inhibiting the SP-NK1R signaling pathway at the cellular level. Likewise, the concentration of BTX-A is positively correlated with its ability to inhibit the metabolism of collagens. Moreover, cotreatment with SP and its inducing agent capsaicin did not reverse the effect of BTX-A in inhibiting the expression of collagens. In summary, these *in vitro* studies have demonstrated that BTX-A inhibits collagen secretion by downregulating the SP-NK1R signaling pathway, thus showing its potential as a therapeutic drug for preventing pathological scars.

To further investigate the feasibility of using BTX-A as a therapeutic drug for hypertrophic scarring, an *in vivo* study was carried out in classic rabbit models. The results confirmed the role of capsaicin in inducing SP in tumorous hypertrophic scars. Pretreatment with different concentrations of BTX-A successfully reduced the thickness of the scar. With a larger dose of BTX-A, this effect became even more significant. Masson trichrome staining demonstrated a reduction in collagens in the dermis when treated with higher doses of BTX-A (4 U/mL, 10 U/mL) at the end of 3 weeks. However, this difference slowly faded and disappeared by the end of 6 weeks. Therefore, we considered that the effect of early BTX-A treatment focused on inhibition of collagen secretion instead of collagen deposition. PCNA showed a regression of cell migration in the BTX-A group, which is identical to the HE staining results. It is worth mentioning that even after pretreatment with high-dose capsaicin, which eventually led to SP overexpression, the thickness and protein level of the scar as well as cell migration did not exhibit a significant increase. Hence, we confirmed that the powerful effect of BTX-A in inhibiting fibroblast metabolism is independent of the local SP concentration. In summary, BTX-A could become an effective therapeutic drug for the early treatment of hypertrophic scars.

Despite demonstrating the promising effect of BTX-A on the SP-NK1R pathway in hypertrophic scars, our study also has several limitations. Specific SP antagonist treatment is needed to verify the effect of SP on fibroblast proliferation. The binding of BTX-A with SP or NK-1R has yet to be discovered. Apart from the SP-NK1R pathway, other possible mechanisms of action of BTX-A in inhibiting hypertrophic scars need to be explored. Multiple pathways such as TGF-β/Smad, TGF-β/MMPs and BMP4/Smad were reported to be mediated by BTX-A in the regulation of collagen (41–43). Furthermore, BTX-A has also been proved to reduce the secretion of pro-inflammatory factors by targeting SNAP23, SNAP25 and TLR2/MyD88 signaling (44, 45). Thus, the interactions between SP-NK1R and the other pathways remain unclear. In the present study, we proved that the SP-NK1R pathway plays an important role in scar formation by stimulating fibroblasts. BTX-A treatment can restrain the effect of this pathway and thus inhibit scar formation. These findings indicate that inhibition of CNI by early injection of BTX-A, resulting in a decrease in collagen deposition, may represent a promising novel therapeutic target for the treatment of hypertrophic scars.

## Data Availability Statement

The raw data supporting the conclusions of this article will be made available by the authors, without undue reservation.

## Ethics Statement

The studies involving human participants were reviewed and approved by Independent Ethics Committee of Shanghai Ninth People's Hospital affiliated to Shanghai Jiao Tong University School of Medicine. The patients/participants provided their written informed consent to participate in this study. The animal study was reviewed and approved by Animal Experimentation Ethics Committee of the School of Medicine, Shanghai Jiao Tong University.

## Author Contributions

SZ, KL, ZY, and JC performed the conduction of experiments and statistical analysis. SZ and KL were major contributors in writing the manuscript. PM and ZZ conceived and designed this study and revised the manuscript. YZ revised the manuscript. All authors read and approved the final manuscript.

## Funding

This study was supported by the National Natural Science Foundation of China, Award Number: 81801918.

## Conflict of Interest

The authors declare that the research was conducted in the absence of any commercial or financial relationships that could be construed as a potential conflict of interest.

## Publisher's Note

All claims expressed in this article are solely those of the authors and do not necessarily represent those of their affiliated organizations, or those of the publisher, the editors and the reviewers. Any product that may be evaluated in this article, or claim that may be made by its manufacturer, is not guaranteed or endorsed by the publisher.
