# Supplementary material for: Dramatic Effect of Botulinum Toxin Type A on Hypertrophic Scar: A Promising Therapeutic Drug and Its Mechanism Through the SP-NK1R Pathway in Cutaneous Neurogenic Inflammation
Source: Front Med (Lausanne). 2022 Mar 3;9:820817. doi: 10.3389/fmed.2022.820817 (PMC8927735; doi:10.3389/fmed.2022.820817)
Supplement: Supplementary file 1 [file Table_1.DOCX]

**Supplementary Table 1: Patient Demographics**

| Patient | Sex | Age (years) | Clinical Diagnosis |
| --- | --- | --- | --- |
| 1 | Male | 35 | Hypertrophic Scar |
| 2 | Female | 28 | Hypertrophic Scar |
| 3 | Female | 32 | Hypertrophic Scar |
| 4 | Male | 9 | Hypertrophic Scar |
| 5 | Female | 15 | Hypertrophic Scar |
| 6 | Female | 29 | Hypertrophic Scar |
| 7 | Male | 38 | Hypertrophic Scar |
| 8 | Male | 33 | Hypertrophic Scar |
| 9 | Female | 24 | Single Eyelid |
| 10 | Female | 38 | Lower Eyelid Bags |
| 11 | Female | 27 | Single Eyelid |
| 12 | Female | 30 | Single Eyelid |
| 13 | Male | 43 | Lower Eyelid Bags |
| 14 | Female | 22 | Single Eyelid |
